# Threshold-Based Random Charging Scheme for Decentralized PEV Charging Operation in a Smart Grid

**DOI:** 10.3390/s17010039

**Published:** 2016-12-26

**Authors:** Ojin Kwon, Pilkee Kim, Yong-Jin Yoon

**Affiliations:** School of Mechanical and Aerospace Engineering, Nanyang Technological University, 50 Nanyang Avenue, Singapore 639798, Singapore; ojinkwon518@gmail.com (O.K.); PKim@ntu.edu.sg (P.K.)

**Keywords:** decentralized charging algorithm, electric vehicles, plug-in electric vehicle, overhead reduction, reduction of charging participation, smart grid, threshold

## Abstract

Smart grids have been introduced to replace conventional power distribution systems without real time monitoring for accommodating the future market penetration of plug-in electric vehicles (PEVs). When a large number of PEVs require simultaneous battery charging, charging coordination techniques have become one of the most critical factors to optimize the PEV charging performance and the conventional distribution system. In this case, considerable computational complexity of a central controller and exchange of real time information among PEVs may occur. To alleviate these problems, a novel threshold-based random charging (TBRC) operation for a decentralized charging system is proposed. Using PEV charging thresholds and random access rates, the PEVs themselves can participate in the charging requests. As PEVs with a high battery state do not transmit the charging requests to the central controller, the complexity of the central controller decreases due to the reduction of the charging requests. In addition, both the charging threshold and the random access rate are statistically calculated based on the average of supply power of the PEV charging system that do not require a real time update. By using the proposed TBRC with a tolerable PEV charging degradation, a 51% reduction of the PEV charging requests is achieved.

## 1. Introduction

Due to environmental and sustainability concerns, the development and the commercialization of plug-in electric vehicles (PEVs) have attracted researchers during the last decade [1,2]. Nevertheless, the penetration of PEV charging systems in the conventional power distribution system may negatively affect the power distribution network in terms of voltage drops and power losses [2,3,4]. Moreover, as the charging of the PEVs is uncoordinated in the existing power distribution network, the penetration of the PEVs will eventually become a burden for the conventional network [5,6]. To optimize the PEV charging system with coordination from a variety of perspectives, several studies have been performed to analyze the possible impacts on the power systems by the PEV charging loads [7,8,9,10,11,12,13,14,15,16]. The main objectives of those studies are the mitigation of the negative impacts by minimizing the increment of peak loads [7,8,9], reducing the power losses [10,11], and minimizing the charging costs [12,13] or maximizing the discharging profits, i.e., vehicle-to-grid [7,14,15,16]. Therefore, most of the research done so far mainly focuses on the centralized optimization of the PEV charging system by stabilizing the power distribution system or by minimizing the charging costs. Recently, decentralized charging schemes have been considered to alleviate the computational complexity of a central controller [17,18,19]. To decide the participation of PEV charging, these previous decentralized charging schemes also require that each customer points of charge (CPOC) should know real time information of all other CPOCs and the central controller, such as a battery state of charging (BSOC), voltage drops and power network constraints. However, communication networks to exchange the real time information among the CPOCs are hard to implement, because the real time information of all the CPOCs and the complexity of the central controller exponentially increase according to the increment of the PEVs in the PEV charging system. As the real time information increases, the cost of data transmissions increases and a complex communication infrastructure is required. For the practical implementation of the decentralized EV charging system, the decentralized charging system should exploit limited information, such as the information of the CPOC itself and statistical information of the power network constraints.

In wireless communication, a selective multiuser diversity (SMUD) scheme is proposed for attaining a reduction in the feedback overhead as well as for a multi-user diversity gain [20,21,22,23,24,25,26]. In the SMUD scheme, users with a channel condition higher than a predetermined threshold, transmit their channel information to a base station, while the others do not transmit. The predetermined threshold is obtained by the average signal-to-noise ratio (SNR), which is related to the distance between the transmitter and receiver. The base station, then, prioritizes the requesters to allocate limited resources for data transmission. Thus, as a portion of users can participate in the requests for the data transmission to the base station, it is possible to reduce the participating users to require the data transmission compared to the conventional multiple user diversity schemes. Therefore, the SMUD can obtain a multi-user diversity and mitigate computational complexity of the central controller. For this reason, the SMUD is widely known as a distributed scheduling algorithm. According to the number of antennas, different types of fading channels and further feedback overhead mitigation, there have been several different types of SMUD. As the base station and mobile users had a single antenna, the threshold was determined by the normalized average SNR [20]. In [21,22], the base station and the mobile users had multiple antennas. In addition, capacity analyses of systems with multiuser diversity for Rayleigh and Nakagami fading channels were researched [23,24]. To further mitigate the feedback overhead, the imperfect average SNR feedback schemes using one bit of information were addressed [25,26]. However, the characteristics of the average SNR for calculating the threshold differ from those of the BSOC in the PEV charging system.

In this paper, a threshold-based random charging scheme (TBRC) is proposed to reduce the number of PEVs participating in the charging requests to minimize the computation complexity of the central controller. As the number of charging requests by the PEVs increase, the handling of the computation complexity in the central controller deteriorates. Thus, only PEVs with a BSOC below the predetermined charging threshold transmit their charging requirements to the central controller, whereas other PEVs with a BSOC above the threshold do not send the charging requests. Because PEVs with a high BSOC cannot receive power for charging the PEVs owing to the power constraints of the distribution network, the charging requests for the PEVs with a high BSOC are dispensable. Therefore, the proposed threshold-based charging method can reduce the number of the charging required PEVs in the PEV charging network. However, most of the PEVs participate in the charging requests at the end of the charging period, because the concentration of the BSOCs in the PEV charging system gradually increases. This indicates that the number of PEVs with the same BSOC becomes larger towards the end of the charging period. Since the threshold is determined by quantizing the BSOC levels, the number of PEVs below the threshold increases cumulatively with the charging time, which causes a significant increase of control signaling overhead. To further mitigate the charging requests, a randomized charging scheme is also considered, which PEVs below the charging threshold can transmit their charging requests at a predetermined access rate. Both the PEV charging threshold and the random access rate are statistically determined by the average values of the supply power for charging the PEVs and the distribution of the initial BSOCs of the PEVs. Thus, the calculation of the thresholds and access rates do not need real time information exchange and updates among the CPOCs and between the target CPOC and the central controller. Therefore, the PEV can decide to participate in the charging request based on the statistical information of the PEV charging system. Further, the central controller finally determines the set of charging PEVs under the real time power network constraints for guaranteeing the system stability. The simulation results show that the proposed TBRC scheme accurately predicts the real profiles of the BSOC in the PEV charging system and reduces the overhead of the PEV charging requests.

## 2. Methodology

The maximization of the BSOCs obtained by all the PEVs within the network constraints is identified as the key trade-off for the reduction of the burdens of the PEV charging requests. Therefore, a smart charging system with cooperation (SCSC) [27] that maximizes the total BSOCs of all the PEVs is modeled as the basis of comparisons for the overall performance of the proposed TBRC scheme. In this section, the basic information and the methodology of the SCSC and the TBRC are delineated.

### 2.1. Assumptions

In both the SCSC and the proposed TBRC, the battery capacity of the PEVs is assumed as 20 kWh [27]. It is assumed that all the PEVs are charged at off-peak times of the day, from 10 p.m. to 6 a.m. [27]. According to a previous work [28], a PEV typically charges in Mode 1 (at 4 kW) or Mode 2 (at 8 kW). In this paper, the charging rate is constant at 4 kW (Mode 1). As Mode 1 is the slowest charging mode, it accounts for the worst case of a PEV charging scenario and clearly demonstrates the negative effect of PEV charging system.

To simplify the problem, there will be no interruptions during a charging process (i.e., no additional PEVs would enter the system and none of the PEVs would unplug from the network, within the charging duration). These assumptions allow the study to focus on the objective of determining the effect of the reduction in the number of the PEV charging request by comparing the proposed scheme with the SCSC, with all other conditions equal.

In this paper, two different types of loads are considered; one is residential loads that cannot be delayed, such as household heating, lighting, cooking and entertainment, and the other is PEV charging loads that can be elastically delayed. As the distribution network needs to firstly satisfy the expectations of the residential loads while charging the PEVs, it is necessary to consider the effect of the residential loads on the supply power to the PEVs. In other words, the supply power for charging the PEVs is the subtraction between the supply power from a feeder and the residential loads. Furthermore, due to the time-varying property of the supply power from the feeder and the residential loads, we consider that the supply power for charging the PEVs is modeled with a sinusoidal waveform to simplify the problems. In the previous studies [11,18,27], the supply power for charging the PEVs was assumed to be a convex shape with the maximum power in the middle of the night, owing to the decrement of industrial and household loads. In this study, the supply power for charging the PEVs is modeled as a sinusoidal function incorporated with an additional random component, which is in accordance with existing assumed models [11,18,27]. This mathematical model may also enable us to easily perform some parametric studies on the supply power for charging the PEVs, by adjusting its amplitude and period or by transforming its coordinate. If the measured data of the supply power for charging PEVs is obtained, the supply power model could be replaced to a realistic raw-data model for industrial and real applications. In the following section, different ratios of the supply power from the feeder to the residential loads are considered and discussed in more detail.

### 2.2. Simulation Cases According to the Residual Capacity

Three simulation cases will be analyzed for both the SCSC and the proposed TBRC based on different possibilities of the residual capacity owing to the correlation between the residential loads and the supply power. In the first simulation case there is a balanced demand between the residential loads and PEV loads within the network supply. Hence, the first case describes a scenario where the total supply of the power distribution system meets the total demand of the residential loads and the PEV loads. The second possible simulation is conducted when the residential loads increase and the supply power exclusive of the residential loads is less than the PEV load requirements. The simulation for this case is modeled such that the supply power is only sufficient to meet 80% of the PEV load demand. This simulation case is not suitable to establish the policy of the power distribution system, but we would like to evaluate the feasibility of PEV charging algorithms as a worst case. Finally, a low residential load scenario is also considered. There is extra supply power delivered to PEVs than required. In this case, an over-supply of 20% is modeled in the simulation.

### 2.3. Power Distribution Network and Load Modeling

This section formulates a simulation model with a distribution network and load data broadly based on the previous researches in [16,27]. The total number of PEVs, $N_{total}$, is 1540 charging at different CPOCs [16]. $N_{ch}$ and $T_{ch}$ are the number of charging time-steps and the charging time of each charging time-step used for the simulation, relatively. As a total of 32 time-steps for 8 h of the charging period are considered (from 10 p.m. to 6 a.m.), thus $N_{ch}=32$ and $T_{ch}=1/4$. As there is not real charging data for the PEV charging system, the initial distribution of the BSOCs for all the PEVs (i.e., at 10 p.m. when all the PEVs are first plugged in) is modeled with a normal distribution with the mean (μ) and variance ($\sigma^{2}$) as 10.75 kWh and 6 kWh, respectively [27]. The average total demand energy for the charging of the PEVs [kWh] can be represented by

(1) $${\bar{E}}_{D_{EV}}=\left(BSOC_{\max}-\mu \right)\times N_{total}$$

where $BSOC_{\max}$ is a battery capacity of 20 kWh.

For simulation purposes, the average power supply for charging the PEVs is calculated by the multiplication of different weight factors and the PEV charging demand for the three scenarios. From Equation (1), the average supply energy to charge the PEVs (kWh) which is subtraction between the supply energy from the feeder and the residential load is calculated by

(2) $${\bar{E}}_{S_{EV}}=\omega \times {\bar{E}}_{D_{EV}},$$

(3) $$\omega =\{\begin{array}{cc} 1 & \mathrm{for}\;\mathrm{simulation}\;\mathrm{case}\;1\\ 0.8 & \mathrm{for}\;\mathrm{simulation}\;\mathrm{case}\;2\\ 1.2 & \mathrm{for}\;\mathrm{simulation}\;\mathrm{case}\;3 \end{array},$$

where ω is the weight factor for indicating the condition of the power distribution network.

In Section 2.1, synthetic data for the supply power to charge the PEVs are generated by the sinusoidal waveform which consists of two components; a trend (or mean) component and a random component. Thus, the supply power to charge the PEVs is represented by

(4) $$P_{S_{EV}}(t)={\bar{P}}_{S_{EV}}(t)+\eta {\hat{P}}_{S_{EV}}(t)$$

where ${\bar{P}}_{S_{EV}}(t)$ and ${\hat{P}}_{S_{EV}}(t)$ are the trend component and the random component of the supply power to charge the PEVs relatively, *t* is the *t*-th charging time-slot, and η is a constant value related to the magnitude of the randomness of the supply power for charging the PEVs. The trend component of the supply power for charging the PEVs is represented by

(5) $${\bar{P}}_{S_{EV}}(t)=L_{1}\cos(kt+\tau )+L_{2},$$

where $L_{1}$, *k* and τ are constant values to reflect long-term changes of the PEV charging system and determine the shape of the average power supply for charging the PEVs, and $L_{2}$ is a constant which is the relationship between the supply power from the feeder and the residential load. Therefore, $L_{2}$ is a function of ω, which is represented by

(6) $$L_{2}=\frac{{\bar{E}}_{S_{EV}}}{N_{ch}\cdot T_{ch}}=\frac{N_{total}(BSOC_{\max}-\mu )}{N_{ch}\cdot T_{ch}}\times \omega +L_{3},$$

where $L_{3}$ is a constant value in order that ${\bar{E}}_{S_{EV}}$ corresponds to $\omega \times {\bar{E}}_{D_{EV}}$. Therefore, all these constant values are determined by the statistical values of the supply power for charging the PEVs and are not instantaneously changed. If the statistical characteristics of the PEV charging system may be varied according to seasonal or monthly changes, all constant values of the supply power for charging the PEVs are updated periodically. The random component of the supply power for charging the PEVs, ${\hat{P}}_{S_{EV}}(t)$, represents a real time varying component of the supply power for charging the PEVs to reflect short-term changes of the PEV charging system, which is modeled on a normal distribution with mean and variance as 0 and 1, respectively.

In Mode 1 (4 kW), $P_{CPOC}$ is the maximum charging power for each PEV and $P_{CPOC}=4\;\mathrm{kW}$. The maximum number of PEVs charged per time-step based on the statistical information of the PEV charging network can be represented by

(7) $$N_{\max}(t)={\bar{P}}_{S_{EV}}(t)/P_{CPOC}.$$

### 2.4. Smart Charging System with Cooperation (SCSC)

Compared with the performance of the proposed threshold based charging scheme, in this section, the SCSC [27] based on the assumptions is considered for focusing on the maximization of the PEV charging performance without considering the computational complexity of the central controller. At each time-step, all the PEVs in the grid provide their own BSOC information to the central controller. In this paper, the minimization of the number of the PEVs with the low BSOC is considered as the optimization of the PEV charging system. The instantaneous total demand energy for the PEV charging is represented by

(8) $$E_{D_{EV}}=\sum_{i=1}^{N_{total}}\left(BSOC_{\max}-BSOC_{i}(0)\right),$$

where $BSOC_{i}(0)$ is the initial BSOC information of the *i*-th PEV. As the initial BSOCs of the PEVs and the supply power to charge the PEVs are random variables, $E_{D_{EV}}$ may not be always equal to ${\bar{E}}_{D_{EV}}$. However, the average value of $E_{D_{EV}}$ is approximately equal to ${\bar{E}}_{D_{EV}}$, stochastically. The instantaneous demand power for charging the PEVs during the *t*-th charging time-slot under the network constraints, $P_{D_{EV}}(t)$, can be expressed as

(9) $$P_{D_{EV}}(t)=\sum_{i=1}^{N_{total}}\alpha_{i}\cdot P_{CPOC},\text{ subject to}:\;\alpha_{i}=\{\begin{array}{cc} 0, & \mathrm{if}\;BSOC_{i}(t)=BSOC_{\max},\\ 1, & \mathrm{otherwise}, \end{array}$$

where $BSOC_{i}(t)$ is the current BSOC (kWh) of the *i*-th PEV at the *t*-th charging time-slot, and $1\leq t\leq N_{ch}$. According to the power network constraint, $P_{D_{EV}}(t)$ cannot exceed $P_{S_{EV}}(t)$ as

(10) $$0\leq P_{D_{EV}}(t)\leq P_{S_{EV}}(t),$$

(11) $$\sum_{t=1}^{N_{ch}}T_{ch}\cdot P_{D_{EV}}(t)=E_{D_{EV}}.$$

If the demand power for charging the PEVs does not exceed the maximum supply power of the network, all the PEVs that are plugged into the power distribution network will be charged. If the PEVs’ demand power exceeds the feeder’s maximum supply power, the charging system prioritizes the PEVs with a lower BSOC in order to ensure that no PEV is left with a low BSOC at the end of the charging period. Thus, the number of charging PEVs for the corresponding BSOC at the *t*-th charging time-step can be represented by

(12) $$\{\begin{array}{cc} M(t)=\left\lfloor P_{S_{EV}}(t)/P_{CPOC}\right\rfloor , & \mathrm{if}\;P_{D_{EV}}(t)\geq P_{S_{EV}}(t),\\ M(t)=\left\lfloor P_{D_{EV}}(t)/P_{CPOC}\right\rfloor , & \mathrm{if}\;P_{D_{EV}}(t)<P_{S_{EV}}(t). \end{array}$$

In the central controller, PEVs that are served during this charging time-slot are determined by an objective function [27]. The objective function is decided under network constraints to achieve the fairness of PEV charging system, and is represented by

(13) $$\left\{EV_{1}^{*},\cdots ,EV_{M(t)}^{*}\right\}=\underset{\{EV_{1},\cdots ,EV_{M(t)}\}\subset \mathbb{C}}{\arg\max}\sum_{i=1}^{M(t)}\left\{1-\frac{BSOC_{i}(t)}{BSOC_{\max}}\right\}P_{CPOC},$$

where ℂ is the total PEV set in the charging system, $\mathbb{C}=\{EV_{1},EV_{2}\cdots ,EV_{N_{total}}\}$. From (13), the central controller decides the PEVs that will be served during a charging time-slot to optimize the PEV charging system.

**Figure 1:**
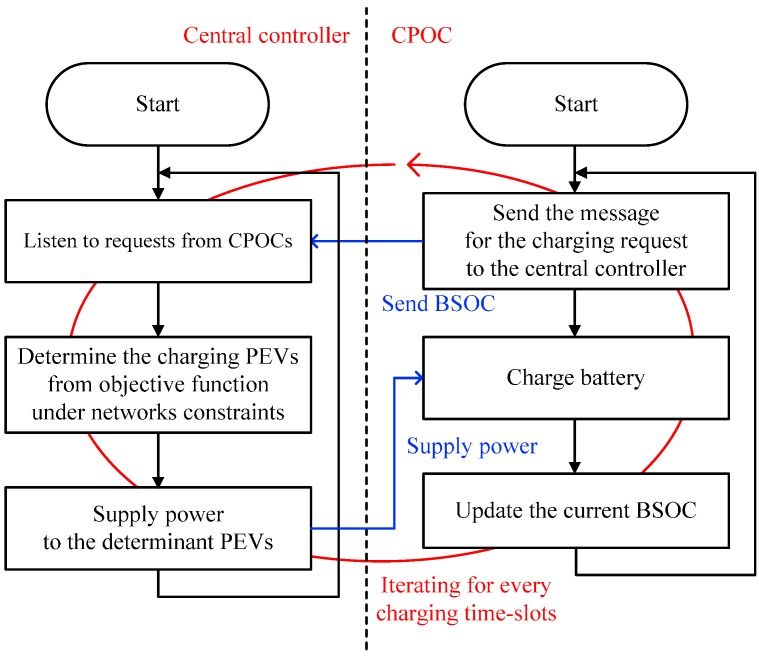
Operational flow of the smart charging system with cooperation (SCSC) for each time-step.

With such a process, the network is coordinated to ensure utilization to its fullest extent in terms of the delivered energy. Under these conditions, in the beginning of every charging time-slot, the BSOCs of all the PEVs are updated and provided to the central controller, repeatedly. In accordance with the condition of the power network such as sudden occurrences of the residual load, the charging PEVs do not fully receive the prearranged charging power in practical. Therefore, significant control signaling overhead related to the current BSOCs of all the PEVs occurs every charging time-slot. The flow chart of the charging process of the SCSC for each charging time-slot is presented in Figure 1.

## 3. Proposed Threshold-Based Charging Scheme Proposed Scheme

**Figure 2:**
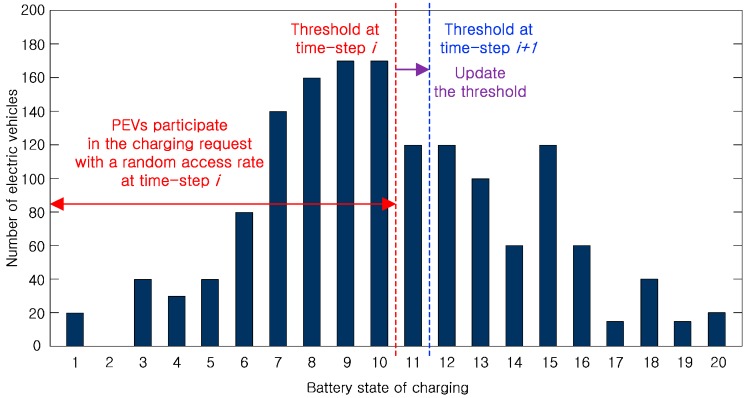
Schematic diagram of the proposed threshold-based random charging (TBRC) scheme.

To reduce the number of the PEV charging participation and the control signal overhead in the centralized charging system, in this paper, an intelligent CPOC for the decentralized charging system is considered with a threshold-based random charging scheme (TBRC) which each PEV decides to participate in the PEV charging request. Figure 2 displays the schematic diagram of the proposed TBRC according to the BSOC. There are 1540 PEVs in the PEV charging system [16] and the distributions of all the PEVs with different BSOCs are shown. The dotted lines indicate the charging threshold value. PEVs with a current BSOC under the predetermined threshold will be charged as per the random access rate, whereas those above the threshold value will not be charged during this charging time-slot.

The predetermined threshold will increase according to the elapse charging time-slot because the average value of the BSOCs of all the PEVs increases owing to the supply power from the distribution network. Thus, the mean of the BSOCs increases, but the variance of the BSOCs decreases according to the elapse charging time-slot. Therefore, instead of the stochastic model, it is necessary to calculate the probability density function (pdf) of the BSOCs according to the charging time-slot by using Table 1. Table 1 shows the number of PEVs for each BSOC at the *t*-th charging time-slot. ${\bar{N}}_{j}(t)$ is the average number of PEVs in the *j*-th level of the BSOC where 0≤j≤20. From the pdf of the initial BSOCs, $N_{\max}(t)$-PEVs with in ascending order of the BSOC are charged under the supply power, ${\bar{P}}_{S_{EV}}(t)$, according to the charging time-slot. Therefore, ${\bar{N}}_{j}(t)$ is sequentially updated by the charged $N_{\max}(t)$-PEVs, and is not a real time changed value because ${\bar{P}}_{S_{EV}}(t)$ is a trend component of the supply power for charging the PEVs.

In this case, γ(t) is the highest charging level of the BSOC at the *t*-th charging time slot, and therefore γ(t) represents the PEV charging threshold at the *t*-th charging time slot. From (7), γ(t) is calculated by

(14) $$\gamma (t)=\arg\underset{\gamma^{*}(t)}{\min}\left(\sum_{j=0}^{\gamma^{*}(t)}{\bar{N}}_{j}(t)\right),\text{ subject to}:\;\sum_{j=0}^{\gamma^{*}(t)}{\bar{N}}_{j}(t)\geq N_{\max}(t),$$

where

(15) $$\begin{array}{cc} N_{\max}(t) & =\frac{{\bar{P}}_{S_{EV}}(t)}{P_{CPOC}}\\ & =\frac{L_{1}\cos(kt+\tau )+L_{3}}{P_{CPOC}}+\frac{N_{total}\cdot \omega \cdot (BSOC_{\max}-\mu )}{P_{CPOC}\cdot N_{ch}\cdot T_{ch}}. \end{array}$$

Thus, Equations (14) and (15) comprise the average values from the statistical data of the PEV charging system and the trend component of the supply power. As the mean and variance of the BSOCs of all the PEVs in the PEV charging system are not changed instantaneously, each CPOC need not update the charging threshold value in accordance with the variations in the instantaneous demand power of the PEVs or the current number of PEVs with the same BSOC. In addition, ${\bar{N}}_{j}(t)\;$ is also not real time changed, because ${\bar{N}}_{j}(t)\;$ is dependent on the pdf of the initial BSOCs. Therefore, each CPOC can calculate the threshold by itself, based on the pdf of the BSOCs according to the charging time-slot before changing the statistical characteristics of the PEV charging system. Table 2 shows a sample of the charging threshold according to the charging time-slot when ${\bar{E}}_{S_{EV}}={\bar{E}}_{D_{EV}}$, $L_{1}$ = 400, *k* = $\frac{2\pi }{T_{ch}}$, τ = π, η=154, $L_{3}$ = 0.

However, in the threshold-based scheme, most of the PEVs participate in the transmission of their BSOC information to the central controller towards the end of the charging period. Because most of the PEVs have similar and high BSOCs owing to a sufficient power supply during the charging period, the concentrated distribution of the BSOCs of the PEVs provokes a significant increment in the requests for the PEV charging. To further mitigate the number of the PEV charging requests, the CPOC randomly participates in the charging request. To calculate the random access rate, the average number of PEVs below the predetermined threshold in the *t*-th charging time-slot is represented by

(16) $${\bar{N}}_{CR}(t)=\sum_{j=0}^{\gamma (t)}{\bar{N}}_{j}(t).$$

From Equation (7), the average maximum number of PEVs served in *t*-th charging time-slot is obtained as $N_{\max}(t)$. Thus, the random access rate is given as

(17) $$\begin{array}{cc} \lambda_{ran}(t) & =\frac{N_{\max}(t)}{{\bar{N}}_{CR}(t)}\\ & =\frac{L_{1}\cos(kt+\tau )+L_{3}}{P_{CPOC}\cdot {\bar{N}}_{CR}(t)}+\frac{N_{total}\cdot \omega \cdot (BSOC_{\max}-\mu )}{P_{CPOC}\cdot N_{ch}\cdot T_{ch}\cdot {\bar{N}}_{CR}(t)}. \end{array}$$

Therefore, Equation (17) is also composed of the average values from the supply power for charging the PEVs.

To further improve the charging performance, the weight factor according to the difference between the current BSOC and the predetermined threshold is considered. Because PEVs with a low current BSOC have a high priority for the charging request to achieve fairness, PEVs that have a lower BSOC far from the charging threshold have a high probability for the charging request. As the *i*-th CPOC knows both the current BSOC and the predetermined threshold, the random access rate of the *i*-th CPOC is represented by

(18) $$\lambda_{ran}^{i}(t)=\lambda_{ran}(t)+\delta \left(\gamma (t)-BSOC_{i}(t)\right),$$

where δ is a weight factor. By using Equations (14) and (18), the CPOC can decide to participate in the charging request to the central controller.

In the case of the central controller, after receiving the PEV charging requests from the CPOCs, $N_{CR}(t)$, the central controller determines the M(t)-PEVs to be served by using an objective function. As $N_{CR}(t)>M(t)$, the optimal serving set composed of the PEVs that require charging is determined by Equation (13). However, as $N_{CR}(t)<M(t)$, all the $N_{CR}(t)$-PEVs can charge at the same time, because $P_{S_{EV}}(t)$ is larger than $P_{D_{EV}}(t)$.

**Figure 3:**
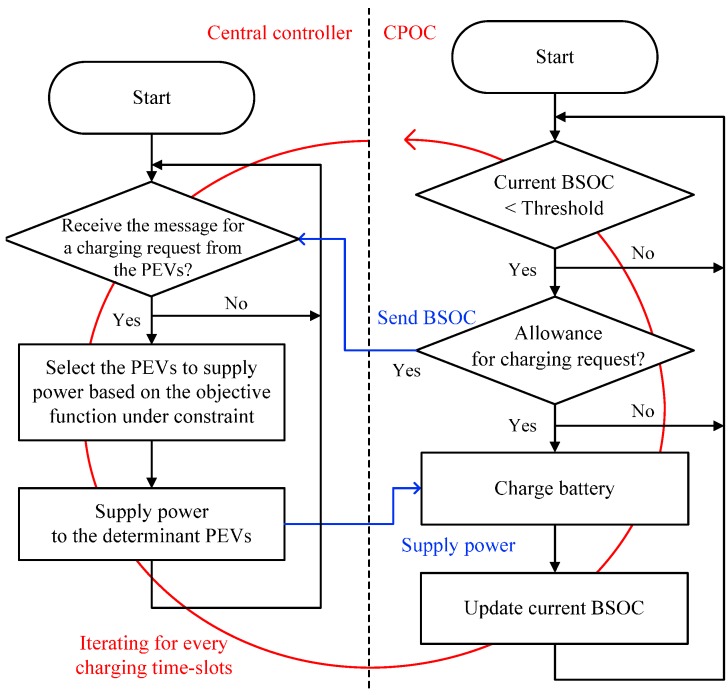
Schematic diagram of the proposed TBRC scheme.

Figure 3 describes the operational flow of the proposed TBRC scheme for a central controller and a CPOC. The central controller receives charging requests from the CPOCs having a BSOC lower than the charging threshold during every charging time-slot. If the demand power for charging requested by the PEVs is larger than the supply power, the central controller selects the charging PEVs under the limitations of the supply power, and then sends the permission and the power to the selected PEVs. As the current BSOC of the PEV is less than the charging threshold, the CPOC opportunistically transmits the charging request to the central controller by considering the predetermined random access rate of Equation (18). Further, the CPOC waits for receiving the permission and the supply power from the central controller. When it receives permission, the CPOC starts to charge the PEV and updates the current BSOC of the PEV. Periodically, the CPOC updates the charging threshold from the central controller after changing the statistical characteristics of the PEV charging system.

Through this process, the proposed TBRC can achieve the reduction of the PEV charging requests based on the decentralized algorithm in which the smart CPOCs individually decide to send the PEV charging request. Different from the conventional decentralized schemes [17,18,19], in the proposed TBRC, the predetermined threshold and random access rate, which are the decision rules in the participation of the PEV charging request, are obtained by the statistical data such as the total number of the PEVs in the system, the mean and variance of the initial BSOCs of all the PEVs, and the average supply power for charging the PEVs. Therefore, the proposed TBRC does not need the exchange of real time information among the CPOCs and the central controller but long term update of the threshold and the access rate when the statistical characteristics of the PEV charging system may be varied according to seasonal changes. Furthermore, the reduction of the PEV charging requests means the diminution of the control signaling overhead among the CPOCs and the central controller.

## 4. Simulation Results

The objective of this simulation is to verify the impact of the proposed scheme on the charging performance and the reduction in the number of PEVs participating in the charging requests at the end of charging period. To manifest the benefits of the optimization technique, the synthetic data of the initial BSOCs is generated using different demand supply scenarios. Table 3 shows the summary of the simulation environments.

### 4.1. Comparison of the Charging Performances of the SCSC and the Proposed Scheme

**Figure 4:**
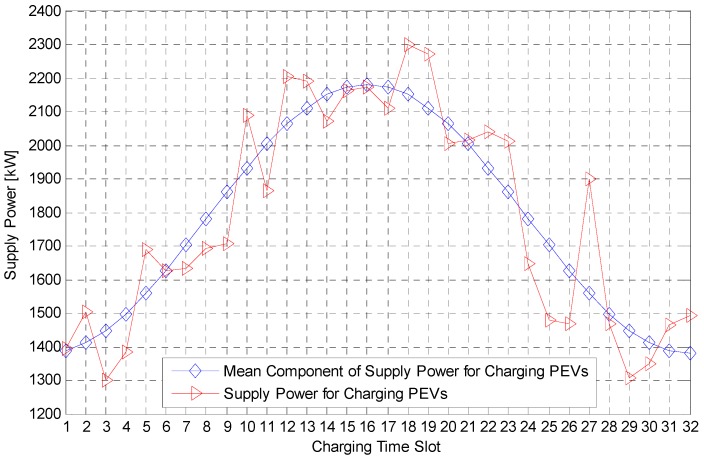
Supply Power for charging the PEVs according to the charging time slot. $P_{S_{EV}}(t)$ is the sum of ${\bar{P}}_{S_{EV}}(t)$ and the random component of the supply power to reflect a real PEV charging environment.

Figure 4 describes the supply power for charging the PEVs from Equation (2). Dotted line shows ${\bar{P}}_{S_{EV}}(t)$ in Equation (5), and solid line shows $P_{S_{EV}}(t)$ that consists of real time supply power. In this simulation, both the threshold and the random access rate are calculated by the average supply power for charging the PEV, ${\bar{P}}_{S_{EV}}(t)$, and the performance of the PEV charging system is simulated by $P_{S_{EV}}(t)$.

**Figure 5:**
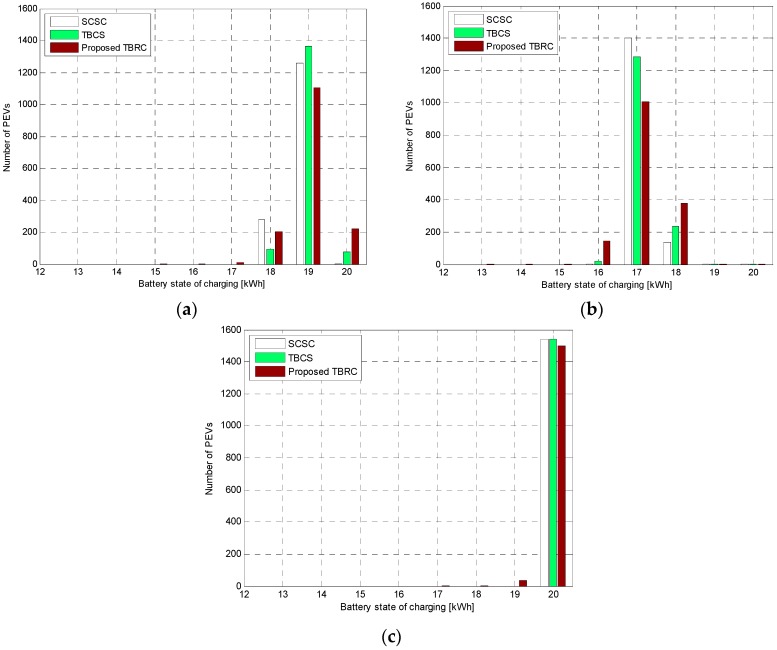
Overall Trends of the BSOCs across the Charging Period for the SCSC, the TBCS, and the Proposed TBRC according to the Different Simulation Scenarios. (**a**) Simulation Case 1; (**b**) Simulation Case 2; (**c**) Simulation Case 3.

In Figure 5, the overall trends of the BSOCs for the SCSC, the threshold-based charging scheme (TBCS), and the proposed threshold-based random charging scheme (TBRC) across the charging period are considerably close to each other. Hence, in the proposed TBRC and the TBCS schemes, the charging thresholds statistically determined by the average values of the supply power for charging the PEVs are proven to be appropriate. However, the charging performance of the proposed TBRC scheme at the end of charging period is lower than those of the other schemes because of the random access method.

In simulation case 1, (${\bar{E}}_{S_{EV}}={\bar{E}}_{D_{EV}}$), all the PEVs that are plugged into the distribution network are almost fully charged (ranging from 17 to 20 kWh) at the end of the charging period for the SCSC. Meanwhile, in the proposed TBRC scheme, the distribution of the BSOCs of the PEVs is approximated to that of the SCSC but is slightly less than that of the SCSC. In the proposed TBRC scheme, 99.40% of the PEVs achieve a high BSOC ranging from 17 to 20 kWh and only 0.60% of the PEVs have a BSOC from 15 to 16 kWh.

In simulation case 2, (${\bar{E}}_{S_{EV}}=0.8{\bar{E}}_{D_{EV}}$), all the PEVs cannot fully be charged because ${\bar{E}}_{S_{EV}}<{\bar{E}}_{D_{EV}}$. Similar to simulation case 1, the overall charging performances of the TBCS and the proposed TBRC scheme are lower than that of the SCSC. However, 9.44% of the PEVs have a BSOC of 16 kWh, and only 0.29% of the PEVs are located on the range from 13 to 15 kWh. Actually, the simulation case 2 is not supposed to happen in the real power distribution system, but it is considered as the worst case for the power supply of the PEV charging system.

In simulation case 3, (${\bar{E}}_{S_{EV}}=1.2{\bar{E}}_{D_{EV}}$), in the proposed TBRC scheme, 100% of the PEVs have their BSOCs within the range 17–20 kWh at the end of the charging session. The overall result is considered satisfactory for such a scenario. Therefore, irrespective of the simulation cases, the charging performances of the proposed charging schemes are lower than that of the SCSC, but the differences in the performances between the proposed schemes and the SCSC might be tolerable.

### 4.2. Comparison of the Control Signaling Overhead

Table 4 shows the comparison of the number of the PEVs participating in the charging requests for the SCSC, the TBCS, and the proposed TBRC scheme. In the proposed TBRC scheme, the average total number of the participated PEVs in charging, which sends the charging request to the central controller, decreases by 60.34%, 68.15%, and 51.56% for the three different scenarios compared to that of the SCSC, relatively, because in the proposed TBRC scheme only the PEVs having a BSOC below the predetermined threshold can participate in transmitting their charging requests at a predetermined random access rate to the central controller. Therefore, the simulation results demonstrate that the proposed scheme can reduce the participation of the PEV charging request and the control signaling overhead [29].

**Figure 6:**
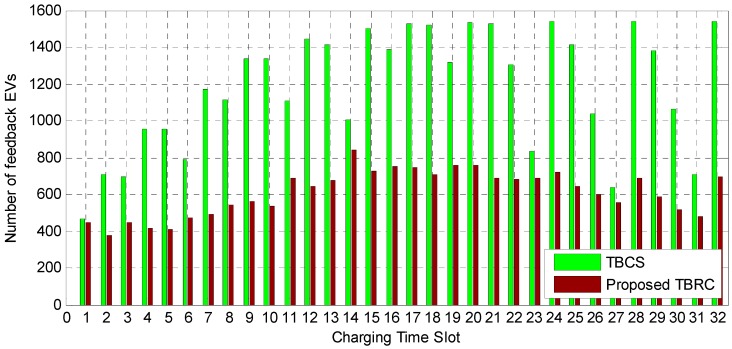
Distribution of the number of PEVs participating in the charging requests according to the charging time-slots (in simulation case 2). In the SCSC, all the PEVs (1540 PEVs) transmit their charging requests to the central controller.

Figure 6 displays the number of charging requests in the entire PEV charging system according to the charging time-slots. In the TBCS, the number of participating PEVs cumulatively increases, because the number of PEVs with the BSOC level near the threshold becomes larger towards the end of the charging period. Thus, the control signal overhead is proportionally increased by the charging requests of the PEVs. In addition, in the SCSC, all the PEVs send their BSOC information to the central controller. Therefore, in the proposed TBRC scheme, the number of PEVs participating in the charging requests is less than those of the SCSC and the TBCS.

## 5. Conclusions

In the coordinated PEV charging system, computational complexity of a central controller is significantly large enough to optimize the PEV charging system based on real time information of all PEVs and power network constraints. To resolve this problem, in this paper, a new threshold-based charging operation with a random access rate is proposed to minimize the number of PEVs participating in the transmission of the charging requests. To reduce the participation of the PEVs in the charging requests, only PEVs below the predetermined charging threshold can have the opportunity of transmitting their charging requirements at the predetermined access rate. Different from conventional decentralized schemes, both the charging threshold and the random access rate are statistically calculated based on average values of the supply power for charging the PEVs. Thus, the proposed TBRC does not share real time information among different CPOCs and a central controller, and needs to update the threshold and the access rate at long intervals.

In computer simulations, the proposed TBRC scheme was found to reduce the participation of the PEV charging requests up to 60.34% of that required in the coordinated PEV charging scheme, when the supply power is equal to or more than the demand power in the proposed TBRC scheme. In this case, the charging performance degradation of the proposed TBRC is negligible. Therefore, the proposed TBRC scheme has shown a substantial reduction in the control signaling overhead and a satisfactory performance of PEV charging system in the simulation cases.

For all simulation cases, we adopted a simple power distribution network with the least practical constraints to clearly demonstrate the difference between the present TBRC and the SCSC and also to avoid heavy computation. However, for the industrial and real-world applications of the proposed TBRC, it is firmly believed that the practical constraints of a distribution network (e.g., over/under voltage, feeder congestion, transformer capacity, and three-phase unbalance) need to be further considered.

## Figures and Tables

**Table 1:** Number of plug-in electric vehicles (PEVs) and the Corresponding battery state of charging (BSOC) at the *t*-th Charging Time Slot.

BSOC	0	1	⋯	Charging BSOC γ(t)	⋯	20
Number of PEV ${\bar{N}}_{j}(t)$	${\bar{N}}_{0}(t)$	${\bar{N}}_{1}(t)$	⋯	${\bar{N}}_{\gamma (t)}(t)$	⋯	${\bar{N}}_{20}(t)$

**Table 2:** Sample of the Charging Threshold according to the Charging Time-slots.

Time-Slot	1	8	16	24	32
Threshold [BSOC]	10	13	15	17	20

**Table 3:** Simulation Environment.

Simulation Parameters	Values
Number of PEVs	1540
Charging hour	10 p.m. to 6 a.m.
Number of charging time-slot	32
Interval of charging time-slot	15 min
Charging rate	4 kW
Battery capacity of the PEVs	20 kWh
Initial distribution of the BSOCs for all the PEVs	Normal distribution (μ: 10.75 kWh, $\sigma^{2}$: 6 kWh)
Distribution of the supply power for charging the PEVs	$L_{1}$ = 400, *k* = $\frac{2\pi }{T_{ch}}$, τ = π, η=154, $L_{3}$ = 0
Weight factor (δ)	0.025
Iteration	3000

**Table 4:** Performance Comparison of the SCSC, the Threshold-based Charging Scheme (TBCS), and the Proposed Threshold-based Random Charging Scheme (TBRC).

% of PEVs at 17–20 kWh	Charging Performance (%)			Reduction in the Participating PEVs (%)		
	SCSC	TBCS	TBRC	SCSC	TBCS	TBRC
Simulation Case 1	100	100	99.40	0	23.26	60.34
Simulation Case 2	99.73	98.64	90.27	0	28.34	68.15
Simulation Case 3	100	100	100	0	22.96	51.56
